# Enhanced plasmonic behavior of bimetallic (Ag-Au) multilayered spheres

**DOI:** 10.1186/1556-276X-6-279

**Published:** 2011-04-04

**Authors:** Ovidio Peña-Rodríguez, Umapada Pal

**Affiliations:** 1 Institut de Ciència de Materials de Barcelona (ICMAB-CSIC), Campus UAB, Bellaterra, Barcelona, 08193, Spain; 2 Instituto de Física, Benemérita Universidad Autónoma de Puebla, Apartado Postal J-48, Puebla, Puebla 72570, México

## Abstract

In this article we study the plasmonic behavior of some stable, highly biocompatible bimetallic metal-dielectric-metal (MDM) and double concentric nanoshell (DCN) structures. By simply switching the material of the inner structure from Au to Ag, the intensity of their surface plasmon resonance could be increased in the optical transparency region of the human tissues up to 20 and 60 percent for the MDM and DCN, respectively, while the biocompatibility is retained. The obtained results indicate that these novel structures could be highly suitable for surface enhanced Raman scattering and photothermal cancer therapy.

## Background

Surface plasmon resonance (SPR), which comes from the collective oscillation of conduction electrons, dominates the optical spectra of metallic nanoparticles (NPs), making them attractive for many potential applications. For example, the dependence of their SPR frequency on the dielectric constant of embedding medium [1] can be used for cancer treatments [2,3], and biological or chemical sensing [4-6]. Moreover, the intense electromagnetic fields produced by the SPR in the surroundings of the nanoparticle are essential in surface-enhanced Raman scattering (SERS) [7], which in turn has important applications in areas such as medical diagnostics [8] and immunoassay [9,10].

Traditionally, gold and silver have been the preferred materials for the synthesis of nanoparticles [11-14], and both of them have some advantages and disadvantages. Gold NPs are easier to synthesize, have better biocompatibility and long-term stability but silver NPs have a more intense SPR, which is of great advantage for SERS and sensing applications. On the other hand, it can be attractive [15] to use bimetallic NPs, where the advantages of both materials can be combined to obtain structures with improved optical response. Nevertheless, while it has been demonstrated [15] that three-layered nanoshells of SiO_2_-Au-Ag can theoretically improve their optical response, those structures present some practical problems related to the difficulty of maintaining separate gold and silver layers, as they tend to form alloys [16-18].

Multilayered nanoshells or nanomatryushkas are a new kind of particles which have been synthesized recently [19-21], and studied theoretically [22-25]. In addition to their better SPR tunability, there appears no problem of alloying in such nanostructures as the metallic layers are separated by dielectric ones. In spite of this potential advantage, there exists no systematic study in literature on the advantages of replacing monometallic multilayered structures with bimetallic ones.

In this work, we have used classical Mie calculations [26-28] to study two different kinds of bimetallic multilayered nanoshells: metal-dielectric-metal (MDM) structures and double concentric nanoshells (DCNs). The bulk values of Ag and Au dielectric functions reported by Johnson and Christy [29] were used to calculate their optical responses after applying the usual size correction [30]. It has been observed that the configurations containing silver at the inner layer and gold at the outer are particularly advantageous as the SPR intensity can be increased without compromising biocompatibility and stability of the nanoparticles. Obtained results have been explained in terms of the theory of plasmon hybridization. These bimetallic structures could be used as excellent replacements for monometallic ones in most sensing and SERS-based applications.

## Procedure

**Figure 1:**
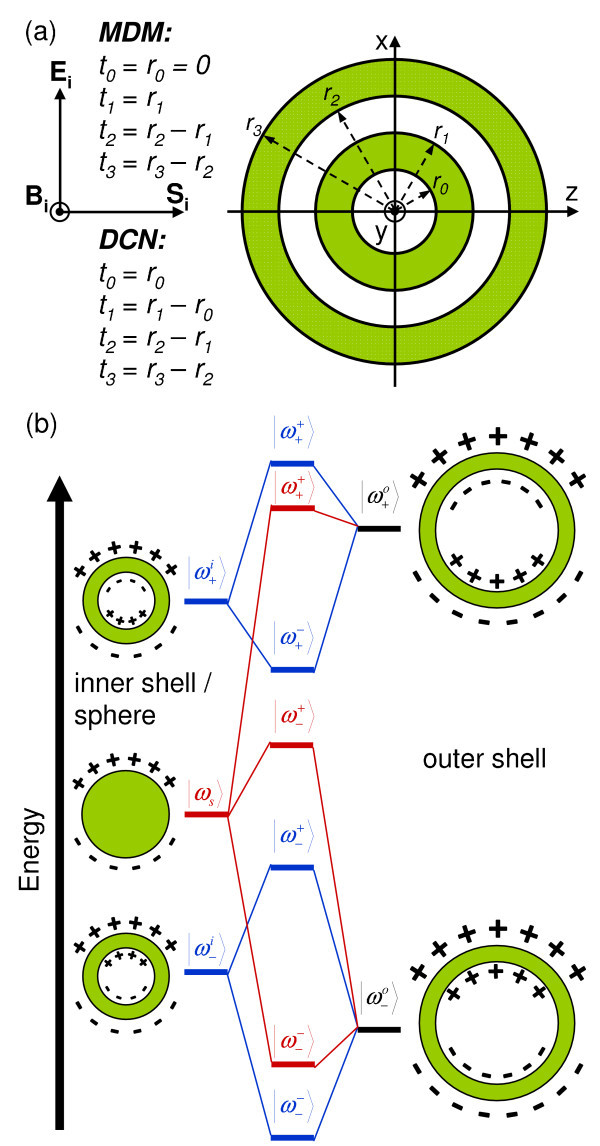
**Schematic representation of the MDM and DCN structures**. **(a) **Schematic representation of the studied MDM and DCN structures (for the MDM *r*_0 _= *t*_0 _= 0) and **(b) **their corresponding energy diagrams, representing plasmon hybridization.

In the present article, we have studied bimetallic MDM structures and DCNs with geometries as shown in Figure 1a, where *r_i _*and *t_i _*(*i *= 0, ..., 3) are the radii and thicknesses of the *i*th layer (for the MDM structure *r*_0 _= *t*_0 _= 0). The extinction efficiencies for the different configurations were calculated by means of Scattnlay [28], a computer implementation of the algorithm developed by Yang [31] for the calculation of the scattering of EM radiation by a multilayered sphere. Moreover, in order to explain the observed shifts of the SPR, we have used a complementary, mainly qualitative method which has been developed recently: the theory of plasmon hybridization [20]. In this approach the characteristics of the SPR are explained in terms of the interactions between the plasmons of metallic nanostructures with simpler shapes. For instance, the SPR of metallic nanoshells can be understood from the coupling between the plasmons modes of a sphere (|*ω*_s_〉) and a cavity (|*ω*_c_〉), where two new plasmon oscillation modes are created: a higher energy (antibonding) mode (|*ω*_+_〉) and a lower energy (bonding) mode (|*ω*_-_〉), corresponding to the antisymmetric and symmetric interactions between the |*ω*_s_〉 and |*ω*_c_〉 modes, respectively.

The energy level diagram for plasmon hybridization in the studied MDM (red) and DCN (blue) structures is depicted in Figure 1b. The plasmon resonance in a MDM [DCN] structure can be viewed as the interaction between the plasmon responses of the inner sphere [nanoshell] (|*ω*_s_〉 [ and ]) and the outer ( and ) nanoshell. Three hybridized modes are obtained for the MDM, the energy mode  corresponds to the antisymmetric coupling between the symmetric plasmon resonance modes of the outer () nanoshell and the sphere plasmon. The coupling between the higher-energy antibonding mode of the outer nanoshell and the nanosphere plasmon modes is very weak and only one hybridized mode is produced in this case (). In contrast, four hybridized modes are produced in the DCN structure because its inner nanoshell has two energy modes. The energy mode  () corresponds to the antisymmetric (symmetric) coupling between the symmetric plasmon resonance modes of the inner () and outer () nanoshells. On the other hand, the energy mode  () corresponds to the symmetric (antisymmetric) coupling between the antisymmetric plasmon resonance modes of the inner () and outer () nanoshells (Figure 1c). Although, in principle, there exists also a coupling between the antisymmetric and symmetric plasmons of the separate nanoshells, it has only a small influence on the hybridized modes, due to the large energy separation between those two modes [20].

The characteristics of the surface plasmon resonance obtained for the multilayer structures studied in this work primarily depend on the properties of the SPR of the two constituents metal layers (their composition and thickness) and the strength of the coupling between them (defined by the thickness of the dielectric layer that separates them). Since the dependence of the optical response in terms of geometrical parameters has been previously studied in detail for both structures [22,23], we focus only on the influence of the composition. For this, we have selected a configuration where both the dielectric layer thickness and the total size of the particles are constant, while the thickness of the metal layers is simultaneously varied (inversely), in order to obtain an opposite behavior between the inner and outer energy modes (one red-shifts when the other does the contrary). For this configuration, the optical response of the bimetallic nanostructures can be tuned to find an optimum relation between *t*_1 _and *t*_3 _yielding the maximum gain in intensity for a given red-shift. Moreover, the optical response can be analyzed across the full range where it can vary in a single simulation. Calculated values were compared with those obtained for the equivalent Au-only structures in both cases.

## Results and discussion

**Figure 2:**
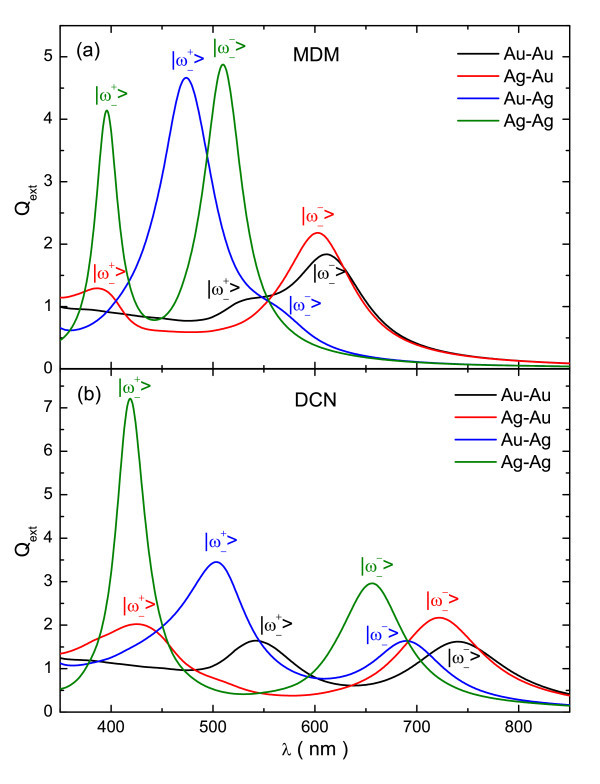
**Simulated extinction efficiency for MDM and DCN structures of various compositions**. Simulated extinction efficiency as a function of the wavelength for **(a) **MDM and **(b) **DCN structures with a fixed thickness of 5 nm in all of its layers (for the MDM *r*_0 _= *t*_0 _= 0). Black, red, blue, and green lines correspond to the Au-Au, Ag-Au, Au-Ag, and Ag-Ag compositions, respectively.

The wavelength variations of extinction efficiency (*Q*_ext_) for MDM and DCN structures of different compositions with a fixed thickness of 5 nm for all of its layers and embedded in water (*n *= 1.33) are shown in Figure 2. It can be seen that, with respect to the equivalent Au-only structures, the Au-Ag and Ag-only configurations have a more intense  energy mode, but the  mode is considerably less intense and blue-shifted. Additionally, the last two configurations are not advantageous for practical applications, due to the poor stability and biocompatibility of silver. On the other hand, the Ag-Au configuration with a more intense  energy mode retains the advantages of Au structures, due to outer Au layer. The only drawback of the latter structures is a slight blue-shift of  energy mode, which could easily be corrected by adjusting their geometrical parameters [22,23]. Considering these findings, we restrict our further studies only to the gains obtained by using the configurations containing Ag and Au at the inner and outer metallic shell, respectively, instead of their Au-only counterparts.

**Figure 3:**
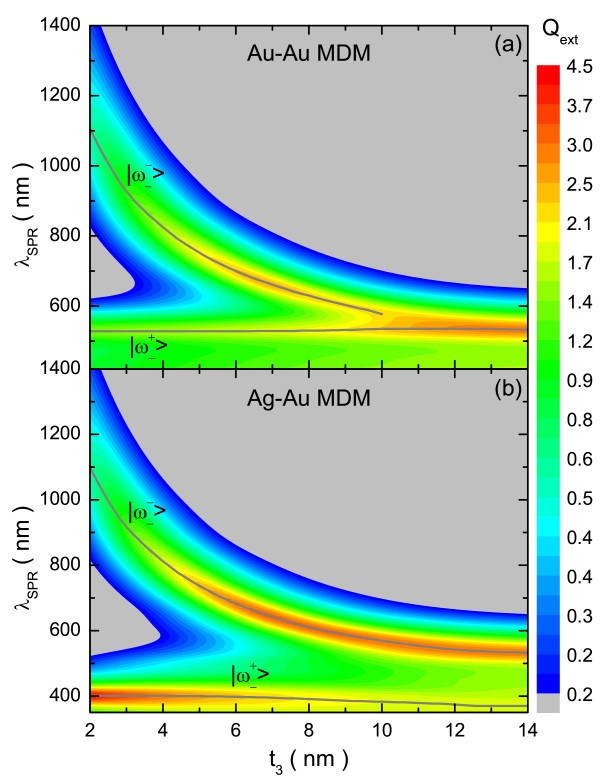
**Simulated extinction efficiency for Au-Au and Ag-Au MDM structures**. Simulated extinction efficiency as a function of the wavelength for **(a) **Au-Au and **(b) **Ag-Au MDM structures, having *t*_2 _= 5 nm, while *t*_1 _and *t*_3 _are varied simultaneously (*t*_1 _= 2, ..., 14 nm; *t*_3 _= 16 nm - *t_1_*).

**Figure 4:**
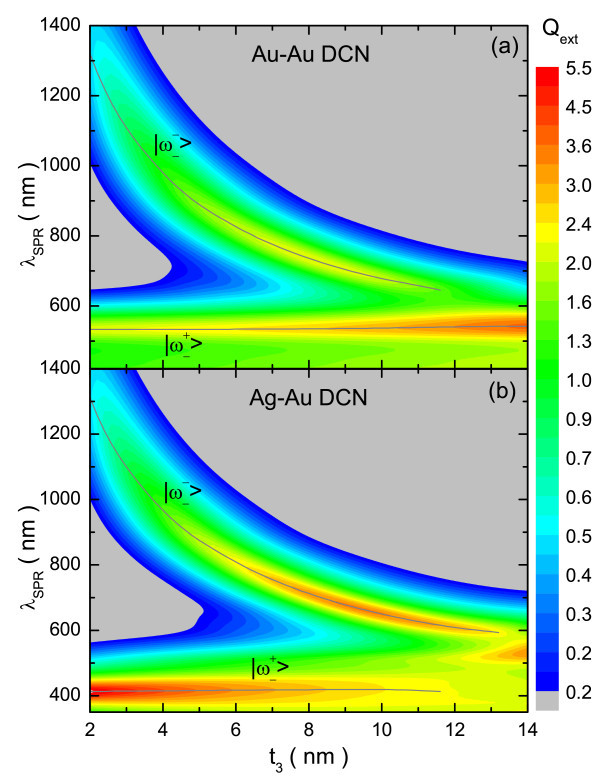
**Simulated extinction efficiency for Au-Au and Ag-Au DCN structures**. Simulated extinction efficiency as a function of the wavelength for **(a) **Au-Au and **(b) **Ag-Au DCN structures, having *t*_0 _= *t*_2 _= 5 nm, while *t*_1 _and *t*_3 _are varied simultaneously (*t*_1 _= 2, ..., 14 nm; *t_3 _*= 16 nm - *t_1_*).

The theoretical extinction efficiencies, calculated for fixed dielectric layers (5 nm) and the simultaneous inverse variations of *t*_1 _and *t*_3 _(*t*_1 _= 2, ..., 14 nm; *t*_3 _= 16 nm - *t*_1_) are shown in Figures 3 and 4 for the MDM and DCN structures, respectively. The spectra obtained as a function of *t*_3 _can be roughly divided into three regions. The first one corresponds to a very thin outer layer (below 5 nm); for which the  and  modes are widely separated. In this case, the position of the bonding (antibonding) mode of the composite structures is almost entirely controlled by the  () mode. For this reason the bonding mode (the one we are interested in) is virtually identical for both compositions and the gain obtained in this case is quite small.

The intermediate region, which includes thicknesses of the outer layer ranging from 5 to 10 nm, is the most interesting because appreciable increases in the intensity of the  mode are obtained for the bimetallic particles, at the expense of a small blue-shift. When both effects are weighted, a net gain of intensity is obtained, revealing the advantage of using bimetallic structures. The improvement in intensity can be explained by considering the characteristics of the geometry as well as the differences between the SPRs of silver and gold. Firstly, in this configuration the internal and external energy modes are closer to each other, resulting in a greater coupling between them, and consequently, both have some influence over the two hybridized modes. Moreover, the SPR of silver is considerably more intense than that of gold, and appears at higher energy (lower wavelength). Thus, the changes in the  mode are driven by the increased influence of the inner metallic layer. Fortunately, the increase in intensity dominates over the red-shift, improving the optical response. On reducing the value of *t*_3_, the  mode red-shifts, getting away from the  mode and reducing their cross-influence over the hybridized modes, and then the differences in the  mode between the Au-Au and Ag-Au configurations are erased (first region).

For the third region (*t*_3 _> 10 nm), again there are few differences between the two compositions; here the bonding hybridized mode keeps blue-shifting until it almost disappears, "absorbed" by the antibonding one. This effect is produced by the shielding caused by the thick outer layer, through which the light does not "see" the inner region and, therefore, the particle behaves almost like a single Au nanoshell.

**Figure 5:**
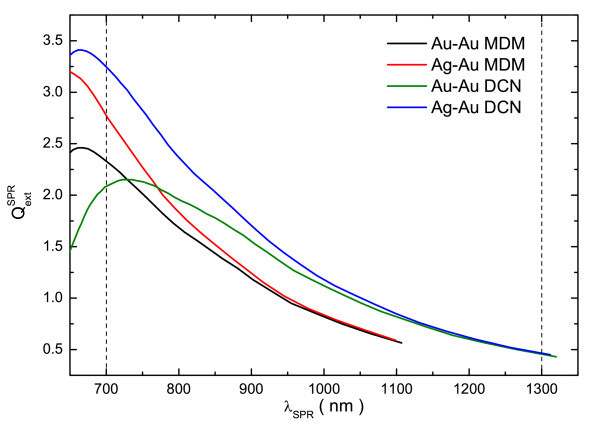
**Summary of the variations of  as a function of *λ*_SPR_**. Summary of the variations of the extinction efficiency at the SPR maximum () as a function of its wavelength (*λ*_SPR_), for the studied MDM and DCN structures. The region of transparency of the human tissues (700-1300 nm) is marked with vertical dashed lines.

Finally, in Figure 5, the variations of the extinction efficiency at the SPR maximum () as a function of its wavelength (*λ*_SPR_), for the considered bimetallic structures are summarized. The region of transparency of the human tissues (700-1300 nm) is marked with vertical dashed lines. As can be seen, in spite of the differences in the intensity and red-shifts, the dependence of the  energy mode on the geometrical parameters is essentially the same for both the MDM and DCN structures. Moreover, the intensity of their SPR bands is greater than the same for monometallic (Au-Au) structures in the 700-1300 nm spectral range. The gain in SPR intensity is particularly important in the region between 700 and 900 nm, which is up to 20 and 60 percent for the MDM and DCN, respectively. Therefore, these bimetallic nanoshell structures could be excellent replacements for the Au-only ones for bio-medical applications such as biosensors and cancer treatments [3].

## Conclusions

By manipulating structural parameters of bimetallic MDM (DCN) structures, a gain in intensity of  mode up to 20 (60) percent can be achieved over their Au-only counterparts in the region of transparency of human tissues. The condition for such gains is that the outer metal layer has a thickness in the range of 5 to 10 nm; in this configuration the internal and external energy modes are closer, so that the interaction between them is greater and, consequently, both have some cross-influence over the hybridized modes. Thus, the  energy mode "inherits" the spectral position of the energy mode of the outer metallic layer and the intensity of the inner one. Our designed bimetallic nanostructures could be more suitable than the conventional mono metallic nanoparticles and nanoshell structures for SERS and cancer therapy applications.

## Abbreviations

DCN: double concentric nanoshell; MDM: metal-dielectric-metal; NPs: nanoparticles; SERS: surface enhanced Raman scattering; SPR: surface plasmon resonance.

## Competing interests

The authors declare that they have no competing interests.

## Authors' contributions

OPR conceived the study, performed the calculations and participated in the analysis of the results and writing the manuscript. UP supervised the study and participated in the analysis of the results and writing the manuscript. All authors read and approved the manuscript.
